# Supplementary material for: Effectiveness and Safety of Palbociclib Plus Endocrine Therapy in Patients with Advanced Breast Cancer: A Multi-Center Study in China
Source: Cancers (Basel). 2023 Sep 1;15(17):4360. doi: 10.3390/cancers15174360 (PMC10487219; doi:10.3390/cancers15174360)
Supplement: Supplementary file 1 [file cancers-15-04360-s001.zip › Supplementary Tables and Figures.pdf]

Supplementary Table S1. Name of participating hospitals

No	Name of participating hospitals
1	the First Affiliated Hospital with Nanjing Medical University
2	Guangdong Provincial People's Hospital
3	The Affiliated Cancer Hospital of Zhengzhou University & Henan Cancer Hospital
4	Guangdong Provincial Hospital of Chinese Medicine
5	Liaoning Cancer Hospital &Institute
6	Union Hospital Tongji Medical College Huazhong University of Science and Technology
7	Tianjin Medical University Cancer Institute and Hospital
8	The First Affiliated Hospital of the University of Science and Technology of China

Supplementary Table S2. Patient Baseline Demographic and Disease Characteristics

Characteristic	Patients (N=397)
BMI *, n (%)	
Underweight	25(6.30)
Normal weight	211(53.15)
Overweight	115(28.97)
Obesity	26 (6.55)
Not reported	20 (5.04)
Methods of castration	
Natural	230 (57.93)
Chemical	131 (33.00)
Surgical	36 (9.07)
Underlying disease	
Yes	88 (22.17)
No	309 (77.83)
Operation mode	
Conserving surgery	27 (6.80)
Modified radical mastectomy	203 (51.13)
Radical mastecomy	22 (5.54)
Total mastectomy	19 (4.79)
Else	20 (5.04)
Unknown	16 (4.03)
Expression of estrogen receptor (ER)	
Negative (<1%)	3 (0.76)
Low-Positive (1%-10%)	8 (2.02)
High-Positive (>10%)	378 (95.21)
Unknown	8 (2.02)
HER2 status	
Positive	26 (6.55)

Weak positive	202	(50.88)
Negative	161	(40.55)
Unknown	8	(2.02)
Ki67 index		
Low (<15%)	73	(18.39)
Mediation (15%-30%)	132	(33.25)
high (>30%)	159	(40.05)
Unknown	33	(8.31)
Prior neoadjuvant therapy, n (%)		
Yes	50	(12.59)
No	250	(62.97)
Not reported	7	(1.76)
Prior adjuvant radiotherapy, n (%)		
Yes	158	(39.80)
No	143	(36.02)
Not reported	6	(1.51)
Adjuvant endocrine regimen		
Tamoxifen	112	(28.21)
Letrozole	63	(15.87)
Anastrozole	27	(6.80)
Exemestane	29	(7.30)
Toremifene	24	(6.05)
Tamoxifen followed by aromatase inhibitors	8	(2.02)
Duration of adjuvant endocrine therapy	249	
Less than 60 months	171	(43.07)
Equal to 60 months	50	(12.59)
More than 60 months	28	(7.05)
Reasons for discontinuation of adjuvant endocrine therapy		
Treatment completed	56	(14.11)
Disease progression	140	(35.26)
Intolerable adverse events	2	(0.50)
Patients' willing	21	(5.29)
Else	24	(6.05)
Unknown	10	(2.52)
Line of chemotherapy before CDK4/6i treatment		
Untreated	188	(47.36)
1L	107	(26.95)
≥2L	99	(24.94)
Unknown	3	(0.76)
Re-operate before CDK4/6i treatment		
Yes	52	(13.10)
No	310	(78.09)
Unknown	35	(8.82)

Re-radiotherapy before CDK4/6i treatment		
Yes	47	(11.84)
No	314	(79.09)
Unknown	36	(9.07)
AI use history		
Yes	205	(51.64)
No	192	(48.36)

* Body mass index (BMI) is a simple index of weight-for-height that is commonly used to classify overweight and obesity in adults. It is defined as a person's weight in kilograms divided by the square of his height in meters (kg/m2). Underweight is a BMI less than 18.5, normal Weight is a BMI from 18.5 to 24.9, overweight is a BMI greater than or equal to 25, and obesity is a BMI greater than or equal to 30.

Supplementary Figure S1. Treatment patterns in Chinese real-world population

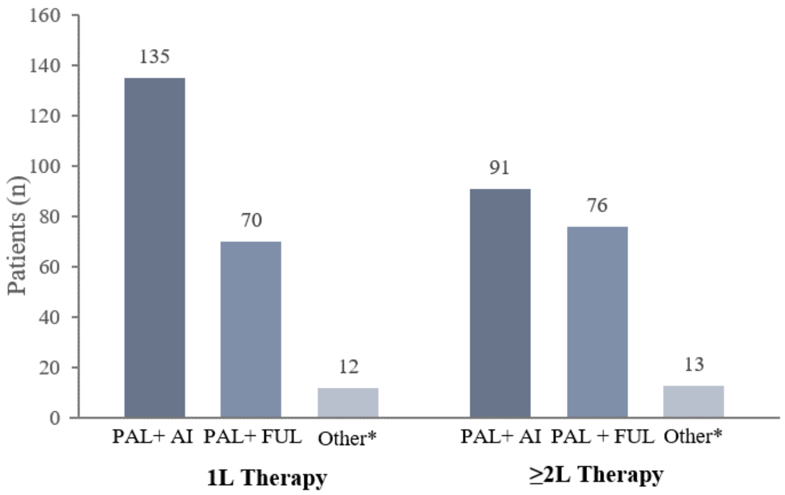

AI, aromatase inhibitors (including letrozole, anastrozole, or exemestane); FUL, fulvestrant.
* Represents patients who, at the onset of the study, received PAL with hormone combinations other than letrozole, anastrozole, exemestane, or fulvestrant or received PAL without hormone combinations.

Supplementary Table S3. Treatment characteristics of Palbociclib

Characteristic	Patients (N=397)	
Combination therapy		
Fulvestrant	148	(37.28)
Letrozole	103	(25.94)
Anastrozole	52	(13.10)
Exemestane	71	(17.88)
Along	17	(4.28)
Else	6	(1.51)
Treatment discontinuation		
Yes	230	(57.93)
Reason for discontinuation		
Disease progression	172	(43.32)

Intolerable adverse events	12	(3.02)
Economy	19	(4.79)
Patients' willing	8	(2.02)
Lost to follow-up	3	(0.76)
Physician's choice	13	(3.27)
Unknown	3	(0.76)

Supplementary Table S4. Univariate analysis for independent factors of PAL

	**All (n = 397)**		**Primary resistance (n = 72)**				**Secondary resistance (n= 231)**			
	**mPFS (mo)**	**P values**	**n (%)**		**mPFS (mo)**	**P values**	**n (%)**		mPFS (mo)	P values
BMI		0.7432				0.6149				0.8147
<24	13.63		43	(59.72%)	9.13		141	(61.04%)	13.43	
≥24	14.17		29	(40.28%)	9.13		90	(38.96%)	14.77	
Underlying disease		0.2832				0.2121				0.4292
Yes	18.77		15	(20.83%)	15.23		53	(22.94%)	17.23	
No	13.50		57	(79.17%)	8.1		178	(77.06%)	13.27	
Menopausal status at study entry		0.2191				0.4217				0.3865
Premenopasual	16.03		20	(27.78%)	8.73		46	(19.91%)	14.5	
Perimenopausal	10.63		7	(9.72%)	5.63		33	(14.29%)	10.33	
Postmenopausal	14.20		45	(62.50%)	9.13		152	(65.80%)	14.23	
Methods of castration		0.3784				0.4225				0.6170
Natural	14.04		36	(50.00%)	9.5		135	(58.44%)	13.5	
Artificial	14.23		36	(50.00%)	5.63		96	(41.56%)	14.23	
ECOG		0.1123				0.0689				0.8584
≤1	14.5		60	(83.33%)	9.33		186	(80.52%)	13.43	
≥2	10.77		12	(16.67%)	6.13		45	(19.48%)	14.77	
Pathology		0.9198				0.5858				0.9546
Invasive ductal carcinoma (IDC)	14.23		33	(45.83%)	9.33		107	(46.32%)	14.2	
Invasive lobular carcinoma (ILC)	14.47		13	(18.06%)	10.3		31	(13.42%)	14.23	
Else	13.43									
Hormone receptor status changes in Secondary pathological		0.6144								
Down-regulation	11.73									
Consistency	13.43									
Up-regulation	12.73									
HER2 status		0.7355								
Positive	13.20					0.3797				0.9282
Weak positive	13.27		40	(55.56%)	8.17		108	(46.75%)	13.27	
Negative	14.23		28	(38.89%)	9.33		102	(44.16%)	13.5	
T		0.2121				0.4272				
T1	11.70		17	(23.61%)	10.3					
T2	13.13		30	(41.67%)	8.73					

T3	Not reached		3	(4.17%)	4.37					
T4	14.23									
N		0.3064		(0.00%)		0.1902				
N0	14.33		11		13.7					
N1	13.13		16	(22.22%)	5.47					
N2	11.73		12	(16.67%)	5.57					
N3	10.73		16	(22.22%)	8.17					
Prior neoadjuvant therapy		0.2603				0.3261				0.7874
Yes	13.27		16	(22.22%)	4.97		31	(13.42%)	14.23	
No	14.33		54	(75.00%)	10.3		191	(82.68%)	13.43	
Prior adjuvant chemotherapy, n (%)		0.1492				0.8690				0.1879
Yes	13.70		53	(73.61%)	8.73		178	(77.06%)	14.5	
No	18.30		18	(25.00%)	11.2		47	(20.35%)	12.1	
Prior adjuvant ET, n (%)		0.4516								0.1202
Yes	13.40						178	(77.06%)	16.03	
No	14.50						20	(8.66%)	10.77	
Duration of endocrine therapy										0.2715
< 5 years							106	(45.89%)	14.23	
5 years							53	(22.94%)	20.37	
> 5 years							38	(16.45%)	12.83	
Adjuvant endocrine regimen		0.8776								
Tamoxifen	14.07		18	(25.00%)	9.13		93	(40.26%)	16.67	
Letrozole	12.83		23	(31.94%)	9.33		41	(17.75%)	12.83	
Anastrozole	21.37		11	(15.28%)	7.03		17	(7.36%)	Not achieved	
Exemestane	16.03		7	(9.72%)	4.8		22	(9.52%)	18.33	
Toremifene	14.20		9	(12.50%)	6.13		19	(8.23%)	18.33	
TAM-AI	11.70						7	(3.03%)	11.7	
Site of metastases										
Lung metastases		0.4565				0.3465				0.7573
Yes	16.67		16	(22.22%)	13.7		89	(38.53%)	13.43	
No	13.40		56	(77.78%)	8.1		142	(61.47%)	14.2	
Brain metastases		0.5245								0.4576
Yes	12.73						21	(9.09%)	12.02	
No	14.23						210	(90.91%)	14.2	
Lymph node metastasis		0.5185				0.8583				0.2035
Yes	13.50		14	(19.44%)	8.17		83	(35.93%)	13.5	

No	14.23		58	(80.56%)	9.13		148	(64.07%)	13.43	
Re-operate before CDK4/6i treatment		0.6250				0.1010				0.1996
Yes	14.77		6	(8.33%)	4.8		39	(16.88%)	16.67	
No	14.20		66	(91.67%)	9.13		178	(77.06%)	13.27	
Dose reduction		0.0640				0.2358				0.4232
Yes	21.37		59	(81.94%)	13.7		30	(12.99%)	16.67	
No	13.43		13	(18.06%)	8.17		201	(87.01%)	13.4	
Adverse events Grade 3/4		0.3260				0.3197				0.5703
Yes	13.70		32	(44.44%)	9.13		118	(51.08%)	14.23	
No	14.23		40	(55.56%)	8.17		113	(48.92%)	13.13	

Supplementary Table S5. The Wilcoxon signed-rank test for the distribution of adverse events in the elderly population and the general population

	Age	N	Median (P25, P75)	Z	P value
Neutrophil count decreased	<65	235	2 (1, 3)	1.116	0.265
	≥65	72	2 (1, 3)		
White blood cell decreased	<65	229	2 (0, 2.5)	1.113	0.266
	≥65	66	2 (1, 3)		
Anemia	<65	95	0 (0, 1)	-1.649	0.099
	≥65	37	0 (0, 1)		
Platelet count decreased	<65	89	0 (0, 1)	-0.31	0.757
	≥65	28	0 (0, 1)		

Supplementary Figure S2. Palbociclib dose adjustment

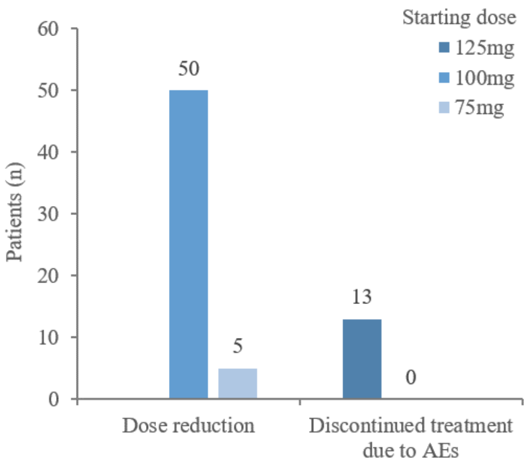

Supplementary Table S6. The X2 test for the relationship between the initial diagnosis stage and line of Palbociclib

	1L (N = 217)	≥2L (N = 180)	P value
Recurrent from earlier stages	154	153	0.001
De novo	63	27	
